# TRPM8 and TRPA1 do not contribute to dental pulp sensitivity to cold

**DOI:** 10.1038/s41598-018-31487-2

**Published:** 2018-09-04

**Authors:** Benoit Michot, Caroline S. Lee, Jennifer L. Gibbs

**Affiliations:** Department of Endodontics, New York University College of Dentistry, New York, USA

## Abstract

Sensory neurons innervating the dental pulp have unique morphological and functional characteristics compared to neurons innervating other tissues. Stimulation of dental pulp afferents whatever the modality or intensity of the stimulus, even light mechanical stimulation that would not activate nociceptors in other tissues, produces an intense pain. These specific sensory characteristics could involve receptors of the Transient Receptor Potential channels (TRP) family. In this study, we compared the expression of the cold sensitive receptors TRPM8 and TRPA1 in trigeminal ganglion neurons innervating the dental pulp, the skin of the cheek or the buccal mucosa and we evaluated the involvement of these receptors in dental pulp sensitivity to cold. We showed a similar expression of TRPM8, TRPA1 and CGRP in sensory neurons innervating the dental pulp, the skin or the mucosa. Moreover, we demonstrated that noxious cold stimulation of the tooth induced an overexpression of cFos in the trigeminal nucleus that was not prevented by the genetic deletion of TRPM8 or the administration of the TRPA1 antagonist HC030031. These data suggest that the unique sensory characteristics of the dental pulp are independent to TRPM8 and TRPA1 receptors expression and functionality.

## Introduction

Dental pulp (DP) is a highly innervated tissue in which sensory afferents have unique characteristics compared to other tissues. In the pulp, although most peripheral nerve endings appear to be small unmyelinated C and small myelinated A delta nociceptors, they originate from large diameter myelinated fibers in the trigeminal ganglion (TG)^1–3^. These sensory afferents have characteristics of low threshold mechanosensors, in that they express NF200, parvalbumin and upregulate NPY after injury^4–6^, but also, a proportion of them express markers of nociceptors such as TrkA, CGRP and TRPV1^3,7^. Further, direct stimulation of the DP, when the protective enamel and dentin are damaged, whatever the modality or intensity of the stimulus, even light mechanical stimulation that would not activate nociceptors in other tissues, produces an intense pain^8^.

In general, detection of thermal and some mechanical stimuli is attributable to receptors of the Transient Receptor Potential (TRP) channel family^9^. Among these receptors, TRPM8 and TRPA1 are important in the activation of sensory neurons by cold temperatures^10,11^. TRPM8 is activated at non-noxious cold temperatures (<25 °C) and clearly contribute to cool temperature perception^10^ whereas TRPA1 is a polymodal receptor activated by noxious cold stimulation (<17 °C), chemical irritants, and appears to be involved in mechanosensation as well^11,12^. Previous studies showed that the pharmacological blockade and genetic deletion of TRPM8 or TRPA1 reduce cold sensitivity in physiological, inflammatory and neuropathic conditions^12–17^.

Although both TRPM8 and TRPA1 are expressed in sensory neurons innervating the DP^18^, their involvement in DP sensitivity to cold and whether they contribute to the sensory characteristics of the DP is, to date, not known. In this study, we examined the expression of TRPM8 and TRPA1 in sensory neurons innervating the DP compared to neurons innervating facial skin or the buccal mucosa. We also evaluated the participation of TRPM8 and TRPA1 in DP detection of noxious cold stimulation.

## Results

### Expression of TRPM8 and TRPA1 in sensory neurons innervating the DP, facial skin or the buccal mucosa

**Figure 1:**
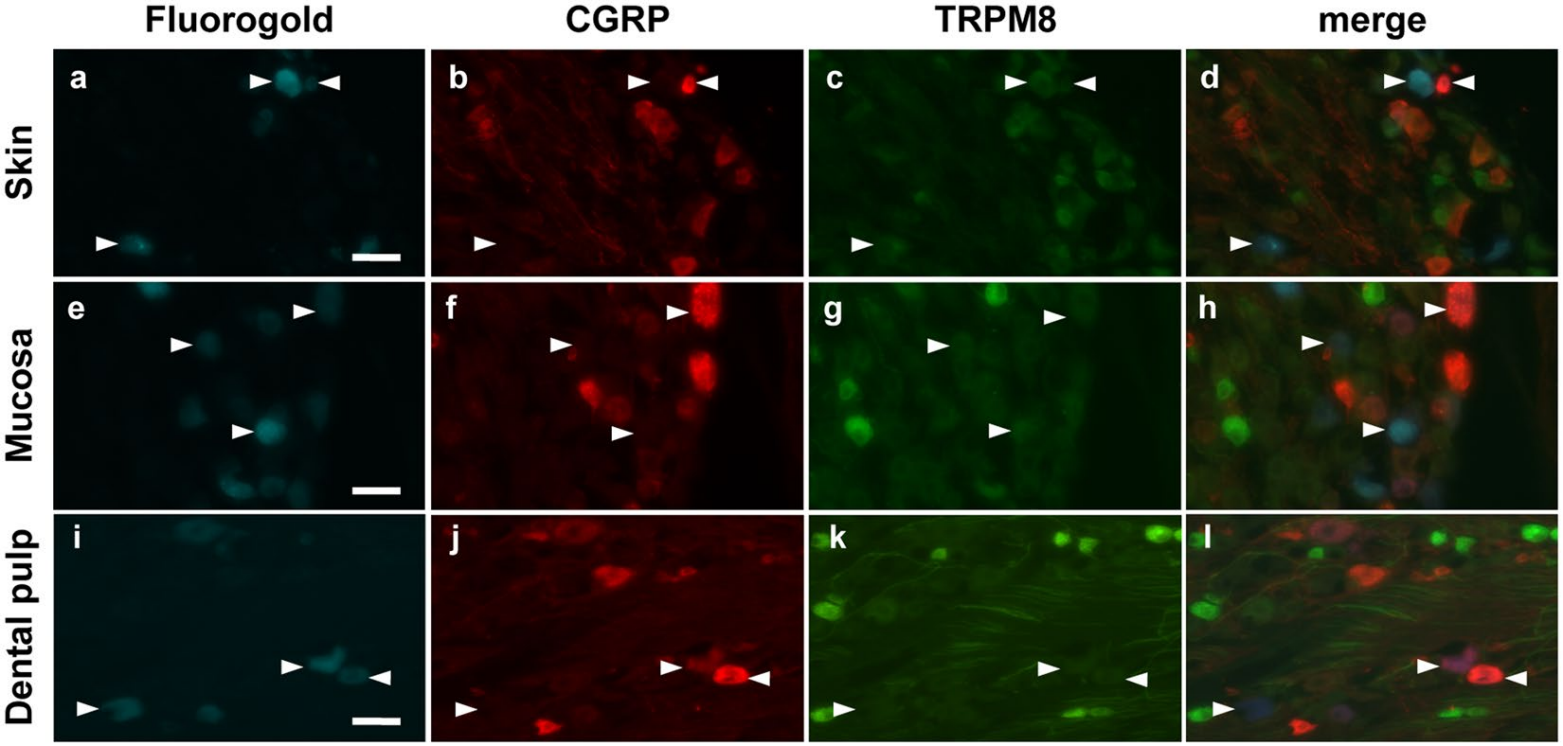
Expression of TRPM8 and CGRP in neurons innervating the facial skin, the buccal mucosa or the DP. Trigeminal sensory neurons innervating either the skin (**a**–**d**), the mucosa (**e**–**h**) or the DP (**i**–**l**) were retrolabeled with Fluorogold (cyan) in *Trpm8*^*tm1Apat*^-GFP-tagged mice. Seven days later TGs were collected and immunostained for CGRP (red). Neurons expressing TRPM8 are stained green. Arrowheads indicate Fluorogold retrolabeled neurons. Scale bar: 50 µm.

**Figure 2:**
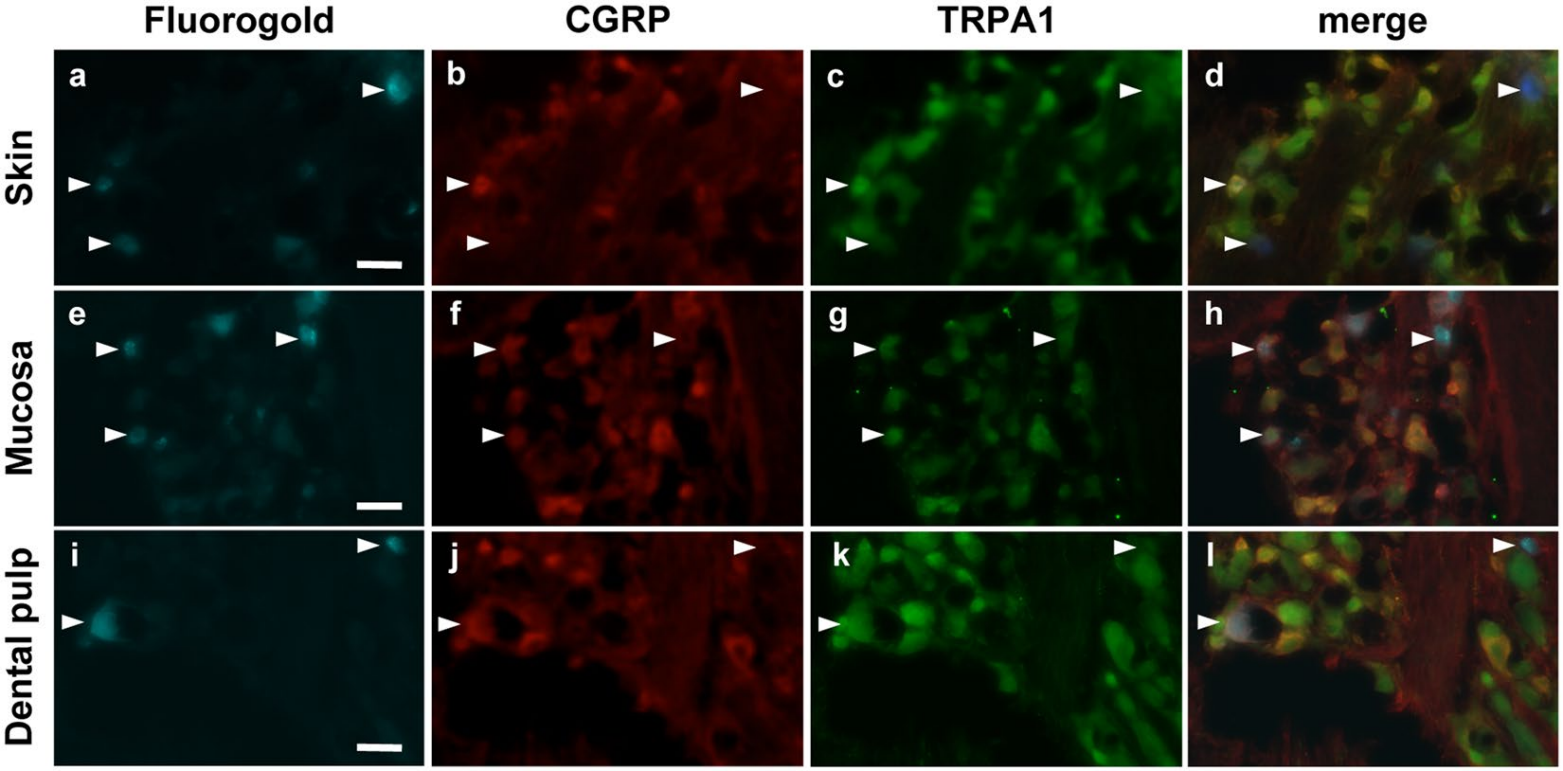
Expression of TRPA1 and CGRP in neurons innervating the facial skin, the buccal mucosa or the DP. Trigeminal sensory neurons innervating either the skin (**a**–**d**), the mucosa (**e**–**h**) or the DP (**i**–**l**) were retrolabeled with Fluorogold (cyan). Seven days later TGs were collected and immunostained for CGRP (red) and TRPA1 (green). Arrowheads indicate Fluorogold retrolabeled neurons. Scale bar: 100 µm

**Figure 3:**
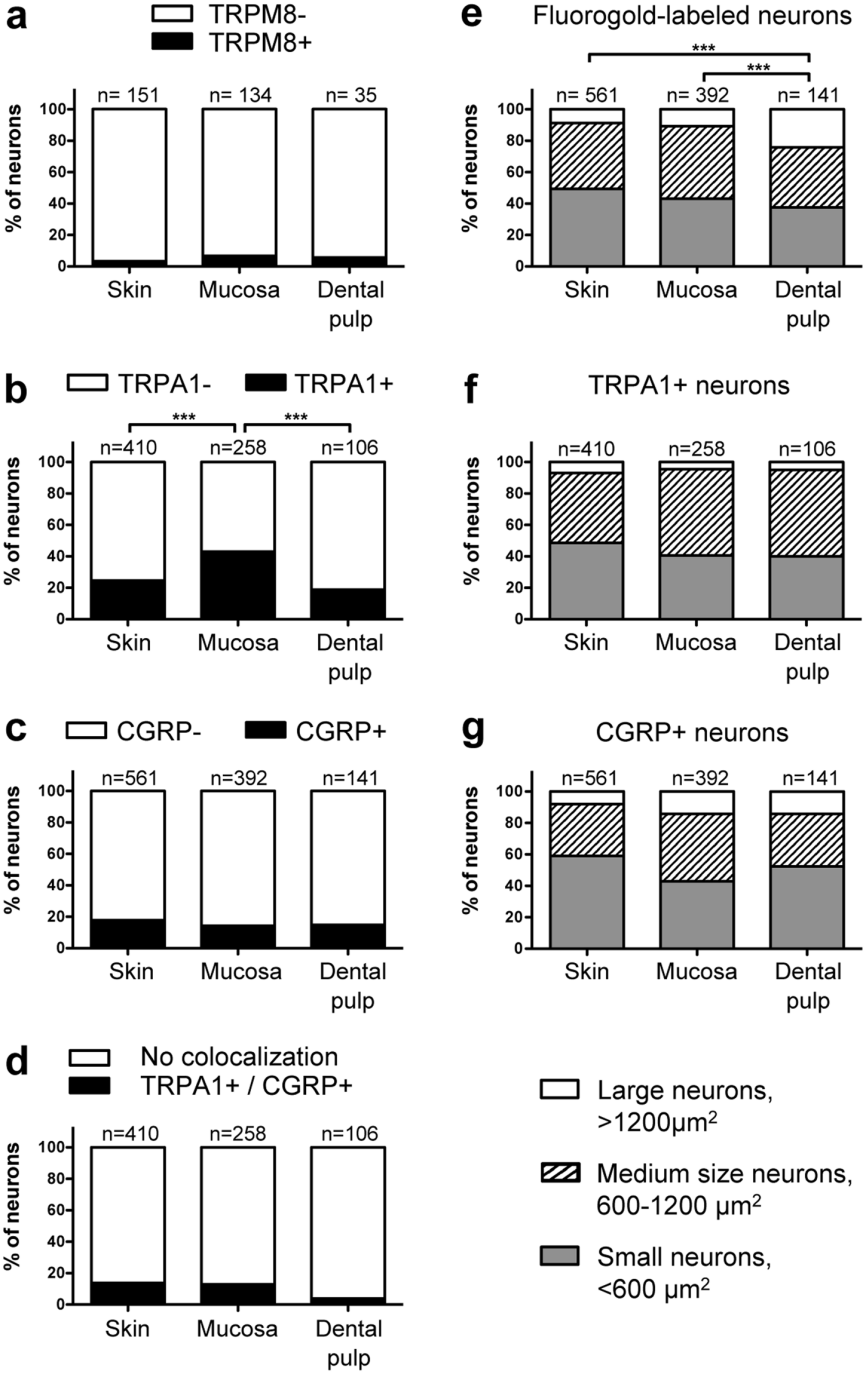
Quantitative analysis of TRPM8, TRPA1 and CGRP expression in trigeminal sensory neurons innervating the facial skin, the buccal mucosa or the DP. Proportion of Fluorogold retrolabeled trigeminal sensory neurons that express, (**a**) TRPM8, (**b**) TRPA1, (**c**) CGRP, and (**d**) both TRPA1 and CGRP. (**e**) proportion of small (<600 µm^2^), medium (600–1200 µm^2^) and large neurons (>1200 µm^2^) in the neuronal population (Fluorogold retrolabeled) innervating either the skin, the mucosa or the DP. (**f**,**g**), proportion of small, medium and large neurons expressing CGRP or TRPA1 in the neuronal population innervating either the skin, the mucosa or the DP. ***p < 0.001, Chi-square test. The number of retrolabeled neurons for each condition is indicated on each histogram. The tissue samples were collected from a total of 6, 7 and 6 animals retrolabeled for skin, mucosa and DP innervating neurons respectively.

The DP has unique sensory characteristics that could be dependent on TRP channel expression in pulp innervating afferents. We addressed this question comparing the expression of TRPM8 and TRPA1 receptors in sensory neurons innervating the DP, the skin of the cheek or the buccal mucosa (Figs 1 and 2). TRPM8 receptors were expressed in 5.7% of neurons innervating the DP which was similar to the frequency of TRPM8 expression in neurons innervating the facial skin and the buccal mucosa (3.3% and 6.7% respectively, Fig. 3a). Similarly, CGRP, a marker of small peptidergic nociceptors, was expressed in the same proportion of labeled sensory neurons whatever the innervating tissue (DP: 14.9%, skin: 17.8% and mucosa: 14.3%; Fig. 3c). Interestingly, TRPA1 was expressed in a higher proportion of neurons innervating the mucosa than neurons innervating the DP (43.0% versus 18.9%, p = 0.0008, Chi-square test; Fig. 3b) or the skin (43.0% versus 24.6%, p < 0.0001, Chi-square test; Fig. 3b). Moreover, we found that a lower, but not statistically significant, proportion of neurons innervating the DP co-expressed TRPA1 and CGRP compared to neurons innervating the skin (3.7% versus 13.7%, p = 0.091, Chi-square test; Fig. 3d) or the mucosa (3.7% versus 12.7%, p = 0.15, Chi-square test, Fig. 3d).

To further characterize neurons innervating the DP we evaluated the size distribution of neurons (Fluorogold positive) innervating the DP, the skin or the mucosa, and neurons expressing TRPA1 and CGRP that innervate each tissue. The proportion of large neurons (>1200 µm2) is significantly higher in the neuronal population innervating the DP (24.1%) than in the neuronal population innervating the skin (8.7%, p < 0.0001, Chi-square test; Fig. 3e) or the mucosa (10.7%, p = 0.0002, Chi-square test, Fig. 3e). The comparison of the size distribution of TRPA1 or CGRP immunoreactive neurons showed no difference between neurons innervating the DP, the skin or the mucosa (Fig. 3f,g).

### Dental pulp sensitivity to cold stimulation is independent to TRPM8 and TRPA1

**Figure 4:**
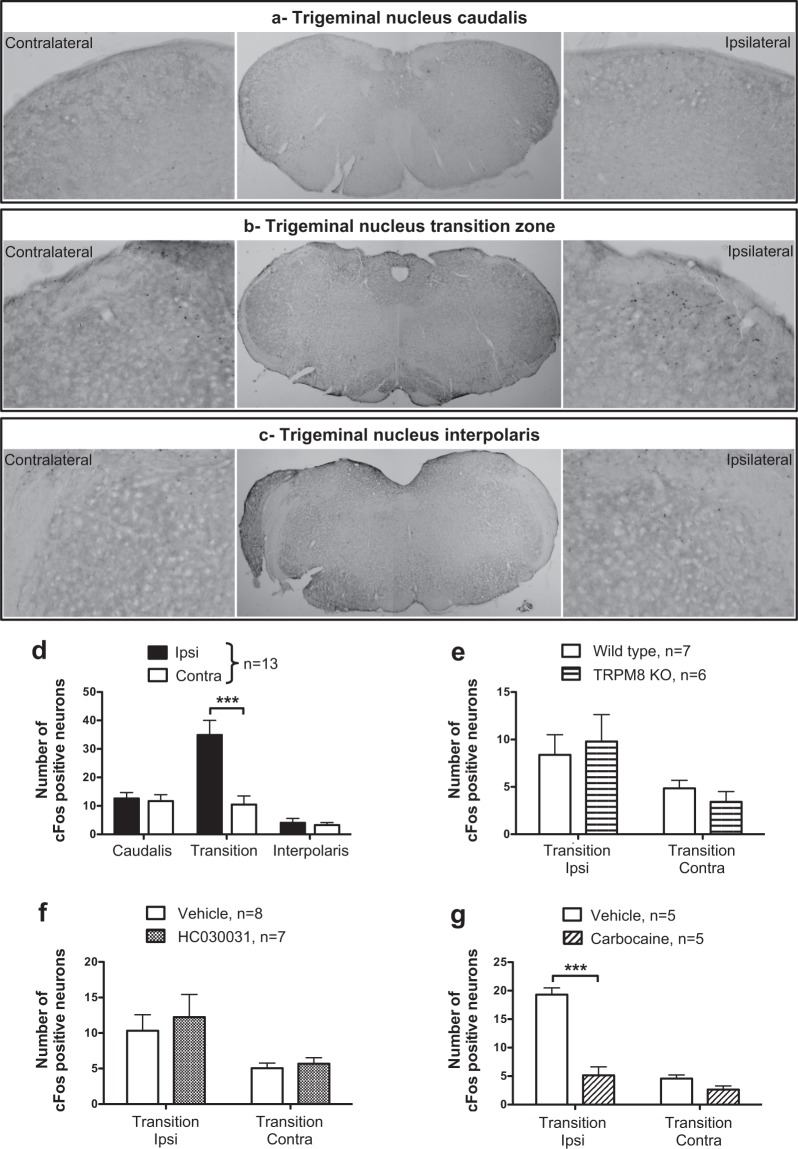
Modulation of cold-induced cFos upregulation in the transition zone of the trigeminal nucleus. Representative photo-micrographs of cFos immunoreactive neurons in the caudalis (**a**), the Vi/Vc transition zone (**b**) and the interpolaris (**c**) regions of the trigeminal nucleus of mice which underwent unilateral cold stimulation of the 1^st^ molar. In (**a**–**c**) the central panel shows a low magnification image of the trigeminal nucleus and the left and right panels are high magnification images of the dorsolateral trigeminal nucleus contralateral and ipsilateral to the stimulation side respectively. (**d**) quantitative analysis of cFos expression in the caudal part, the transition zone and the interpolaris part of the trigeminal nucleus. Effects of genetic deletion of TRPM8 (**e**), HC030031 (TRPA1 antagonist, 100 mg/kg, i.p., administered 1 h before the cold stimulation) (**f**) and Cabocaine (local anesthetic, 3%, injected 10 min before the cold stimulation) (**g**) on cold-induced upregulation of cFos in the Vi/Vc transition zone. Data are expressed as mean + SEM. of the number of neurons/section/animal. ***p < 0.001, Bonferroni’s test, n = 5–13/group.

**Figure 5:**
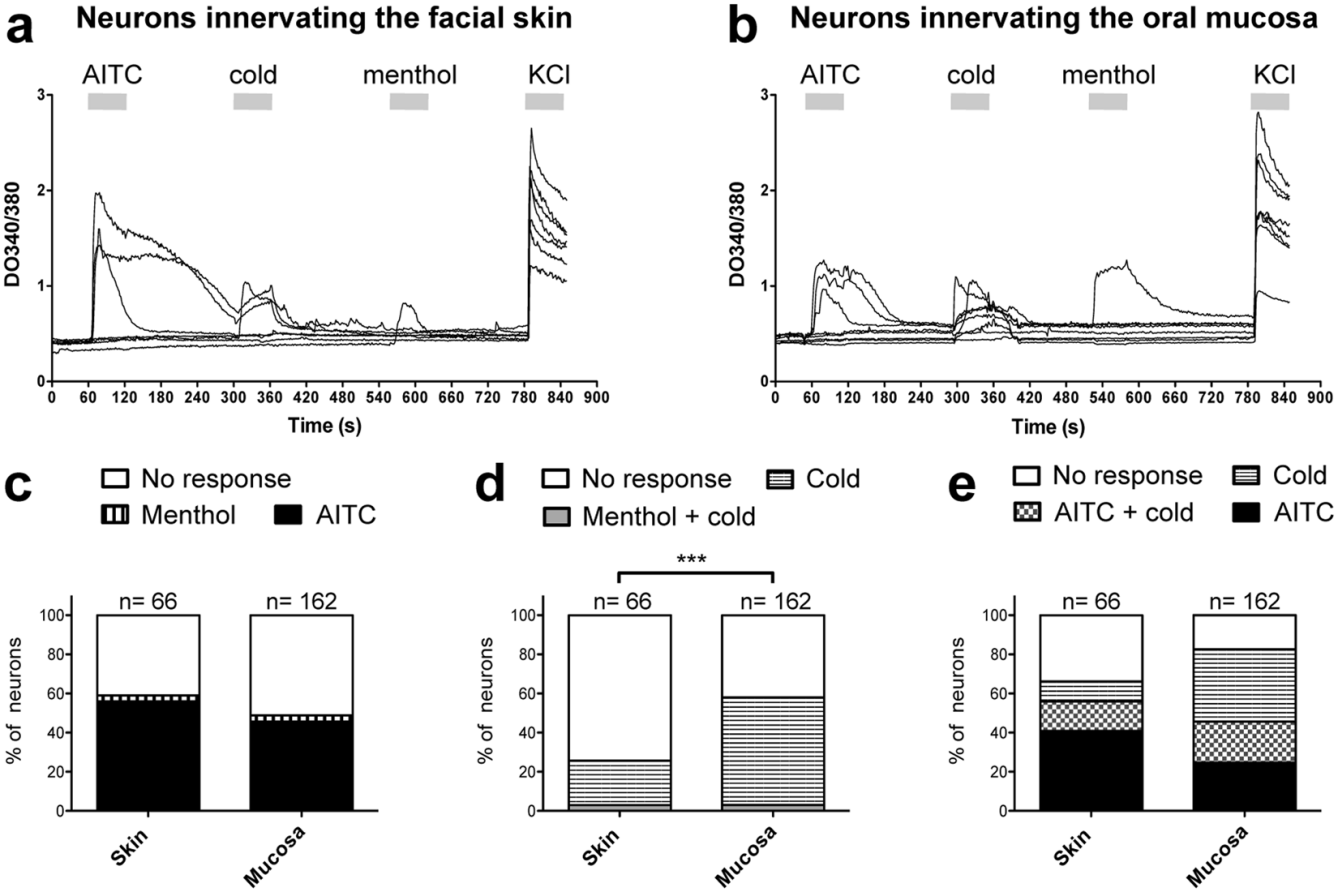
Effects of AITC, cold stimulation and menthol on sensory neurons innervating the facial skin or the buccal mucosa. (**a**,**b**) Each trace represents intracellular calcium levels in one neuron (retrolabeled with DiI) innervating the skin or the mucosa that was treated with AITC (250 µM), cold HBSS buffer, menthol (250 µM), and KCl (75 mM). Horizontal grey bars indicate the duration of each treatment. Bar-graphs show the proportion of neurons responding to, (**c**) AITC or menthol, (**d**) menthol or cold HBSS buffer, (**e**) AITC and/or cold HBSS buffer. Note that no neurons were responsive to both AITC and menthol. ***p < 0.001, Chi-square test, n = 66–162 neurons.

The repeated application of a unilateral cold stimulation on the first maxillary molar induced neuronal activation detected through the increase of cFos expression in the ipsilateral trigeminal nucleus (Fig. 4a–c). In the caudalis and the interpolaris regions of the trigeminal nucleus the number of cFos immunoreactive neurons was not different in the ipsilateral versus contralateral side. However, in the Vi/Vc transition zone between the caudalis and the interpolaris regions, the number of cFos immunoreactive neurons was significantly higher in the ipsilateral compared to the contralateral side (34.8 ± 5.1 and 10.5 ± 3.0 respectively; 2-way ANOVA; side: p = 0.0018, F = 10.6, DF = 1; region: p = < 0.0001, F = 16.28, DF = 2; interaction: p = 0.0002, F = 9.59, DF = 2; Bonferroni’s test, transition zone ipsi versus contra p < 0.001; Fig. 5d). The effects of the unilateral application cotton, free of Frigi-dent, did not induce cFos expression in the ViVc transition zone (Supplementary Fig. S1), indicating that the light mechanical stimulation of the intact tooth without the concomitant cold stimulation, does not induce activation of DP nociceptors.

The involvement of TRPM8 and TRPA1 receptors in DP sensitivity to cold stimulation was evaluated using TRPM8 KO mice and the TRPA1 antagonist, HC030031. In TRPM8 KO mice, cold stimulation increased the number of cFos immunoreactive neurons in the ipsilateral Vi/Vc transition zone (ipsi: 9.8 ± 3.4 versus contra: 3.4 ± 1.1) but the increase was not different to the number of cFos immunoreactive neurons in WT mice (TRPM8 KO ipsi: 9.8 ± 3.4 versus WT ipsi: 8.4 ± 2.1; 2-way ANOVA; side: p = 0.014, F = 7.26, DF = 1; genotype: p = 0.99, F = 0.00001, DF = 1; interaction: p = 0.45, F = 0.59, DF = 1; Fig. 4e). Similarly, the TRPA1 antagonist did not prevent cold-induced upregulation of cFos in the ipsilateral compared to the contralateral trigeminal nucleus (2-way ANOVA; side: p = 0.008, F = 7.99, DF = 1; treatment: p = 0.54, F = 0.37, DF = 1; interaction: p = 0.76, F = 0.096, DF = 1; Fig. 4f). On the contrary, the administration of the local anesthetic carbocaine adjacent to the stimulated tooth reduced the cold-induced increase of the number of cFos immunoreactive neurons in the Vi/Vc region (2-way ANOVA; side: p < 0.0001, F = 65.4, DF = 1; treatment: p < 0.0001, F = 57.3, DF = 1; interaction: p < 0.0001, F = 33, DF = 1; Bonferroni’s test, carbocaine ipsi 5.1 ± 1.5 versus vehicle ipsi 19.3 ± 1.1, p < 0.001; Fig. 4g). This confirms that the transmission of the cold stimulation can indeed be blocked by interfering with peripheral neuronal transmission.

### Effects of Menthol, AITC and cold stimulation on sensory neurons innervating the facial skin and the buccal mucosa

To clarify the participation of TRPM8 and TRPA1 receptor in the detection of cold stimulation, we evaluated neuronal activity in response to ice cooled HBSS (0–2 °C), the TRPM8 agonist menthol and the TRPA1 agonist AITC in neurons innervating the skin or the mucosa. Because neurons innervating teeth are only a low proportion of the total TG neurons we were not able to detect sufficient numbers of DiI retrolabeled sensory neurons innervating the DP.

A similar proportion of neurons innervating the skin or the mucosa responded to menthol (3.0% (2/66 neurons) and 3.1% (5/162 neurons) respectively) or AITC (56.0% (37/66 neurons) and 45.7% (74/162 neurons) respectively, p = 0.10, Chi-square test) and no neurons responded to both menthol and AITC (Fig. 5c). A detailed analysis showed that all neurons responding to menthol were also sensitive to cold stimulation, but a significant proportion of neurons were cold sensitive and menthol insensitive (Fig. 5d). Moreover, compared to the neurons innervating the skin, a higher proportion of neurons innervating the mucosa were cold sensitive (25.7% (17/66 neurons) and 58.0% (94/162 neurons) respectively, p < 0.0001, Chi-square test; Fig. 5d). The analysis of neuronal sensitivity to AITC and cold stimulation showed that a subpopulation of cold sensitive neurons was AITC insensitive (10.6% (7/66 neurons) and 37.0% (60/162 neurons) of neurons innervating the skin or the mucosa respectively; Fig. 5e). Conversely, a significant proportion of neurons innervating the skin (43.9%, 29/66 neurons) or the mucosa (24.7%, 40/162 neurons) were AITC sensitive and cold insensitive, suggesting that TRPA1 is only partially involved in cold perception (Fig. 5e).

## Discussion

In this study, we evaluated DP innervating afferents’ sensitivity to cold though the expression of the cold-sensitive receptors TRPM8 and TRPA1 and their respective contribution to cold-evoked activation of trigeminal neurons. We show that the proportion of TRPM8 and TRPA1 immunoreactive neurons is not different in the neuronal population innervating the DP or the facial skin. Interestingly, a higher proportion of neurons innervating the buccal mucosa were immunoreactive for TRPA1 compared to neurons innervating the other tissues. However, in calcium imaging experiments the selective TRPA1 agonist AITC activated a similar proportion of neurons innervating the skin (56%) compared to neurons innervating the mucosa (47%). Overall, these proportions are higher than what was found in immunohistochemical quantification in which 25% of neurons innervating the skin and 43% of neurons innervating the mucosa were immunoreactive for TRPA1. There are several possible explanations for this difference.

First, the neuronal expression of TRPA1 is highly sensitive to physiopathological conditions, especially inflammation. It was shown that a single subcutaneous injection of formalin or Complete Freund’s Adjuvant increased TRPA1 expression up to one-week post injection^19,20^. Moreover, ethanol, which we used as solvent for the retrotracer DiI, was shown to induce an inflammation associated with cyclooxygenase-2 upregulation in a model of gut inflammation^21,22^ and therefore, could have affected TRPA1 expression in our experiments. Second, although we used an acute culture model to limit the upregulation of TRPA1, the process of dissociating and culturing the TG could produce an upregulation of TRPA1. Barabas *et al*. showed, in cultured mouse TG neurons, a rapid increase in the proportion of neurons responding to the TRPA1 agonist cinnamaldehyde, from 27%, 4 h after cell plating, to 66%, 10 h after cell plating^23^. Thus, the discrepancy between our immunohistochemical versus calcium imaging data could be due to the injection of the retrotracer and the cell culture processing that could have increase TRPA1 expression in TG neurons.

The DP sensitivity to cold was evaluated through the expression of the marker of neuronal activation, cFos, in the trigeminal nucleus. We showed that a repeated unilateral cold stimulation on the tooth increases cFos expression in the ipsilateral trigeminal nucleus compared to the contralateral side. The upregulation of cFos was localized in dorsal part of the Vi/Vc transition zone between the caudalis and the interpolaris regions of the trigeminal nucleus. In general, the projection structure of nociceptive neurons innervating the orofacial area is the caudal part of the trigeminal nucleus^24^. However, it was shown that trigeminal nociceptive neurons project also in the Vi/Vc region and painful stimulation in various orofacial areas, including the DP, activate neurons in the Vi/Vc region^25,26^. The Vi/Vc region is important to trigeminal pain, as a large proportion of Vi/Vc neurons project to the parabrachial nucleus, which is involved in affective and autonomic response to pain^27^. Moreover, a local injection of NMDA receptor antagonist or glial inhibitors in the Vi/Vc region reduced pain induced by temporomandibular joint inflammation^28,29^. Collectively, these data support that the increase in cFos expression in Vi/Vc region observed with cold stimulation of the tooth was a relevant endpoint to assess the participation of TRPM8 and TRPA1 in cold-induced tooth pain.

We showed that the genetic deletion of TRPM8 or the pharmacological blockade of TRPA1 did not reduce the cold-induced cFos upregulation in the trigeminal nucleus, suggesting these receptors have a minor contribution in DP noxious cold perception. Many studies have demonstrated that TRPM8 and TRPA1 receptor mediate cold detection^12,14,16,17,30^, however, in line with our results some contradictory studies using TRPA1 antagonists and TRPA1 KO mice failed to show a reduction of cold sensitivity in physiological conditions^13,15^. Moreover, the lack of TRPM8 contribution in DP cold detection, evidenced by our observation that cold-induced c-Fos upregulation was not prevented in TRPM8 KO mice, would be explained by the small proportion (5%) of neurons innervating the DP that express TRPM8. All together these data suggest that, in physiological condition, DP sensitivity to cold stimuli involves TRPM8/A1 independent mechanisms.

To date the mechanisms underlying the sensory perception of the DP are not fully understood. The most largely accepted theory to explain activation of the pulpal afferents is the hydrodynamic theory, in which tooth stimulation causes fluid movement within dentinal tubules, activating sensory afferents located in or near the dentinal tubules. Indeed, it was shown that application of a noxious cold stimulation (0 °C) on teeth induces dentinal tubule fluid movements and neuronal activation^31,32^. In our experimental condition, the stimulation applied on the tooth was certainly cold enough (Frigi-dent −50 °C out of the can) to induce dentinal tubule fluid movements that would then mechanically activate pulpal sensory afferents activating central pain pathways evidenced by c-Fos upregulation in the trigeminal nucleus.

However, from the study we cannot exclude the participation of an important cold-detecting neuronal population that does not express TRPM8 and TRPA1. In line with our results in sensory neurons innervating the skin and the mucosa, previous studies showed that a significant proportion of sensory neurons are activated by cold stimulation but are insensitive to TRPM8 and TRPA1 agonists^33,34^. The cold sensitive receptors expressed in these neurons have not been fully identified, but could include sodium and potassium channels such as eNaC, TREK1 and TRAAK, which genetic deletion and pharmacological blockade were shown to reduced cold-induced pain^35–37^.

Whether a subpopulation of sensory neurons innervating the DP is indeed cold sensitive but not due to TRPA1 and TRPM8 expression has not yet been investigated and further studies are needed to clarify the potential existence of this neuronal population and the involvement of other cold-sensitive receptors in cold-induced tooth pain.

## Methods

### Animals

All experiments were approved by the Institutional Animal Care and Use Committee at the New York University College of Dentistry and followed the guidelines provided by the National Institutes of Health Guide for the Care and Use of Laboratory Animals. Experiments were performed on 10–12 weeks old male and female C57Bl6 and TRPM8 knock-out (KO) (*Trpm8*^*tm1Apat*^-GFP-tagged) mice purchased from Jackson Laboratory^38^. Animals were housed 2–5/cage in an environment with controlled 12 h/12 h light/dark cycles and had free access to food and water.

### Retrolabeling

Mice were anaesthetized with 2% isoflurane and injected with 2 µl of Fluorogold (2.5% in saline) or 1, 1′-Dioctadecyl-3, 3, 3′, 3′-Tetramethylindocarbocyanine Perchlorate (DiI, 2.5% in ethanol) either intradermally in the skin of the cheek or submucossally in the buccal mucosa to label the respective innervating neurons. Injections were repeated 2–3 times in each region. Retrolabeling of DP innervating neurons was performed in mice deeply anaesthetized with ketamine-xylazine (100-10 mg/kg, i.p.). The enamel of the first molar in each side of the jaw was removed with a ¼ round dental bur at low speed to expose dentinal tubules without exposing the DP to the oral environment. The tooth surface was washed, and the smear layer was removed with 35% phosphoric acid gel. Fluorogold or DiI (1–2 μl) was applied three times with a 5-min drying intervals. At the end of each procedure mice were treated once a day for three days with carprophen (5 mg/kg, i.p.) to avoid the development of a local inflammation.

### Immunofluorescence

Seven days after retrolabeling sensory neurons with Fluorogold, homozygotes and hererozygotes *Trpm8*^*tm1Apat*^-GFP-tagged and wildtype (WT) mice were euthanized and perfused intracardially with 10 ml of phosphate-buffered saline (PBS 1 × ) followed by 30 ml of 10% formalin in PBS. TGs were removed, post-fixed for 30 min in 10% formalin and cryoprotected overnight in PBS with 30% sucrose. TGs were sectioned (16 µm thick sections) with a Cryostat (−20 °C) and collected on superfrost glass slides. Sections were washed for 30 min in PBS, followed by 1 h incubation in a blocking solution (0.3% Triton X100 and 5% normal goat serum in PBS). Then, sections were incubated at 4 °C for either 3 days with rabbit anti-TRPA1 antibody (1:10000; AB58844; Abcam) or 1 day with chicken anti-GFP antibody (1:10000; GFP-1020; Aves Labs). After a 30-min wash in the blocking solution, samples were incubated for 2 h with the secondary antibody Alexa Fluor 488-conjugated goat anti-rabbit (1:700; Life Technology) or Alexa Fluor 488-conjugated goat anti-chicken (1:700; Life Technology). Sections were washed in blocking solution for 30 min and incubated at 4 °C overnight with mouse anti-CGRP antibody (1:8000; C7113, Sigma). The following day, sections were washed for 30 min in PBS and successively incubated for 1 h in the blocking solution and 2 h with the secondary antibody Alexa Fluor 546-conjugated goat anti-mouse (1:700; Life Technology). After a final 30 min wash, sections were coverslipped. Photo-micrographs of TGs were collected with a fluorescent inverted microscope (Nikon Eclipse Ti). The proportion of Fluorogold retrolabeled neurons that expressed TRPA1, GFP and/or CGRP was quantified using the NIH Image J Analysis Software. One representative image of the staining of each protein of interest was selected. We defined, for each selected image, a fluorescent intensity threshold for which all pixels of the image that have a fluorescence intensity higher than the threshold were considered as immunopositive pixels. One fluorescent threshold was defined for each antigen (TRPM8, TRPA1 and CGRP). However, because of the presence of some small artifacts that could be considered as immunostaining, only cells that were stained for more than 50% of the cell body area were considered as immunoreactive cells. Then, each image was analyzed using the corresponding defined threshold, neurons stained for Fluorogold were delineated, the background was subtracted according to the defined threshold, and the number of cells stained for more than 50% of the cell body area was quantified.

### Cold stimulation and cFos immunostaining

Female C57Bl6 mice pretreated with either vehicle, the TRPA1 antagonist HC030031 (100 mg/kg in DMSO, i.p.) or carbocaine (3% in saline, 10 µl injected submucosally in the buccal vestibule adjacent to the stimulated tooth) and TRPM8 KO or WT mice were anaesthetized with ketamine-xylazine. The jaw was opened and a unilateral cold stimulation was applied on the surface of the non-injured intact enamel of the first maxillary molar using a small piece of cotton cooled with Frigi-dent (Ellman International, Inc.). The stimulation was repeated 15 times over a 30-min period. Three hours after the beginning of the stimulation, mice were perfused intracardially with formalin, the brainstem was collected, post-fixed for 3 h and cryoprotected as described in part 2.6. The brainstem was sectioned (40 µm thick sections) using a Cryostat (−20 °C) and collected in containers filled with PBS. Every 5^th^ section was selected for staining. Floating sections were successively incubated for 1 h in a blocking solution (0.3% Triton X100 and 5% normal goat serum in PBS) and overnight with rabbit anti-cFos antibody (1:30000, PC38, Calbiochem). Sections were washed for 30 min with the blocking solution and incubated for 1 h with the secondary antibody biotinylated goat anti-rabbit (1:700; Vector). After a 30-min wash with PBS, the sections were incubated for 30 min in 0.3% H_2_O_2_ in PBS and for 1 h with the avidin-biotinylated-horseradish-peroxidase (ABC Vectastain kit Elite, Vector). Then, they were washed 10 min with PBS and stained black/gray with 3,3′-Diaminobenzidine (DAB kit, Vector). After a final 30 min wash in PBS, sections were mounted on superfrost glass slides and coverslipped. The number of c-Fos immunoreactive neurons was quantified in both ipsilateral and contralateral trigeminal nucleus, in sections from caudalis through interpolaris region. The different regions of the trigeminal nucleus were identified according to the Allen Brain Atlas^39^.

### Trigeminal ganglion neuron culture

TGs from DiI retrolabeled C57Bl6 mice were collected in F12 media, and neuronal culture was prepared as described by Malin *et al*.^40^. Briefly, TGs were cut in ten pieces each and incubated successively for 20 min in papain solution (40units/ml) and for 20 min in collagenase-dispase solution (3.33–4.66 mg/ml). Then, TG neurons were mechanically dissociated with a P200 pipet, suspended in F12 media supplemented with 5% Fetal Bovine Serum and seeded into poly-D-Lysine-coated round glass coverslips. After 2–4 h incubation at 37 °C in a humidified atmosphere containing 5% CO_2_, neurons were processed for calcium imaging experiment.

### Single-cell calcium imaging

TG neurons cultured on coverslips were washed for 10 min in HBSS and loaded in the dark with the fluorescent calcium indicator Fura-2-acetoxy-methyl ester (Fura2, 5 µM) for 45 min at room temperature. Coverslips were washed for 10 min in HBSS and mounted in a microscope chamber. Loaded cells were excited successively (2 Hz) for Fura2 at 340 and 380 nm for 200 ms and emitted fluorescence was monitored at 510 nm using a charged coupled device sensor camera coupled to an inverted Nikon Eclipse Ti microscope. Fluorescence intensities from single cells excited at the two wavelengths were recorded separately, corrected for the background and the fluorescence ratio (F340/F380) was calculated using the software NIS Elements-AR version 4.0. All neurons were stimulated for 1 min successively with the TRPA1 agonist acyl-isothiocyanate (AITC, 250 µM), the TRPM8 agonist menthol (250 µM) and ice cooled HBSS (0–2 °C), with a 3 min wash with HBSS between treatments. At the end of each experiment KCl (75 mM) was applied to identify healthy neurons. Only neurons retrolabeled with DiI that responded to KCl were used for the analysis. A positive neuronal response was defined as a minimum of 20% increase of the ratio F340/F380 relative to the pretreatment F340/F380 value.

### Statistical analyses

Statistical analyses were performed with GraphPad Prism 5 software. Immunostaining of TRPM8, TRPA1 and CGRP, and calcium imaging data are expressed as percentage of immunoreactive or responsive neurons relative to the number of Fluorogold/DiI positive neurons and were analysed with Chi-square test. Results of cFos immunostaining are expressed as a mean + SEM of the number of immunoreactive neurons/side/section/animal and were analyzed with a 2-way ANOVA, followed by Bonferroni post hoc test. The significance level was set at p < 0.05.

## Electronic supplementary material

Supplementary Figures

## Data Availability

The datasets generated during and/or analysed during the current study are available from the corresponding author on reasonable request.

## References

[1] Paik SK (2009). Light and electron microscopic analysis of the somata and parent axons innervating the rat upper molar and lower incisor pulp. Neuroscience.

[2] Fried K, Sessle BJ, Devor M (2011). The paradox of pain from tooth pulp: low-threshold “algoneurons”?. Pain.

[3] Gibbs JL, Melnyk JL, Basbaum AI (2011). Differential TRPV1 and TRPV2 channel expression in dental pulp. J. Dent. Res..

[4] Fried K, Arvidsson J, Robertson B, Brodin E, Theodorsson E (1989). Combined retrograde tracing and enzyme/immunohistochemistry of trigeminal ganglion cell bodies innervating tooth pulps in the rat. Neuroscience.

[5] Itotagawa T (1993). Appearance of neuropeptide Y-like immunoreactive cells in the rat trigeminal ganglion following dental injuries. Arch. Oral Biol..

[6] Ichikawa H (1995). Parvalbumin- and calretinin-immunoreactive trigeminal neurons innervating the rat molar tooth pulp. Brain Res..

[7] Yang H, Bernanke JM, Naftel JP (2006). Immunocytochemical evidence that most sensory neurons of the rat molar pulp express receptors for both glial cell line–derived neurotrophic factor and nerve growth factor. Arch. Oral Biol..

[8] Dababneh RH, Khouri AT, Addy M (1999). Dentine hypersensitivity—an enigma? A review of terminology, epidemiology, mechanisms, aetiology and management. Br. Dent. J..

[9] Julius D (2013). TRP channels and pain. Annu. Rev. Cell Dev. Biol..

[10] Peier AM (2002). A TRP channel that senses cold stimuli and menthol. Cell..

[11] Story GM (2003). ANKTM1, a TRP-like channel expressed in nociceptive neurons, is activated by cold temperatures. Cell.

[12] Kwan KY (2006). TRPA1 contributes to cold, mechanical, and chemical nociception but is not essential for hair-cell transduction. Neuron.

[13] Obata K (2005). TRPA1 induced in sensory neurons contributes to cold hyperalgesia after inflammation and nerve injury. J. Clin. Invest..

[14] Karashima Y (2009). TRPA1 acts as a cold sensor *in vitro* and *in vivo*. Proc. Natl. Acad. Sci. USA.

[15] Chen J (2011). Selective blockade of TRPA1 channel attenuates pathological pain without altering noxious cold sensation or body temperature regulation. Pain.

[16] Knowlton WM (2013). A sensory-labeled line for cold: TRPM8-expressing sensory neurons define the cellular basis for cold, cold pain, and cooling-mediated analgesia. J. Neurosci..

[17] Patel R (2014). Novel TRPM8 antagonist attenuates cold hypersensitivity after peripheral nerve injury in rats. J. Pharmacol. Exp. Ther..

[18] Park CK (2006). Functional expression of thermo-transient receptor potential channels in dental primary afferent neurons: implication for tooth pain. J. Biol. Chem..

[19] Asgar J (2015). The role of TRPA1 in muscle pain and mechanical hypersensitivity under inflammatory conditions in rats. Neuroscience.

[20] Martínez-Rojas VA (2017). Peripheral and spinal TRPA1 channels contribute to formalin-induced long-lasting mechanical hypersensitivity. J. Pain Res..

[21] Kang JY, Teng CT, Wee A, Chen FC (1995). Effect of capsaicin and chilli on ethanol induced gastric mucosal injury in the rat. Gut.

[22] Park S (2000). Capsaicin protects against ethanol-induced oxidative injury in the gastrointestinal mucosa of rats. Life Sciences.

[23] Barabas ME, Kossyreva EA, Stucky CL (2012). TRPA1 is functionally expressed primarily by IB4-binding, non-peptidergic mouse and rat sensory neurons. PLoS One.

[24] Sessle BJ (2000). Acute and chronic craniofacial pain: brainstem mechanisms of nociceptive transmission and neuroplasticity, and their clinical correlates. Crit. Rev. Oral Biol. Med..

[25] Zhou QQ, Imbe H, Dubner R, Ren K (1999). Persistent trigeminal Fos protein expression after orofacial deep or cutaneous tissue inflammation in rats: implications for persistent orofacial pain. J. Comp. Neurol..

[26] Chattipakorn S, Chattipakorn N, Light AR, Narhi M, Maixner W (2005). Comparison of Fos expression within the ferret’s spinal trigeminal nuclear complex evoked by electrical or noxious-thermal pulpal stimulation. J. Pain.

[27] Gauriau C, Bernard JF (2002). Pain pathways and parabrachial circuits in the rat. Exp. Physiol..

[28] Wang H, Wei F, Dubner R, Ren K (2006). Selective distribution and function of primary afferent nociceptive inputs from deep muscle tissue to the brainstem trigeminal transition zone. J. Comp. Neurol..

[29] Shimizu K (2009). Differential involvement of trigeminal transition zone and laminated subnucleus caudalis in orofacial deep and cutaneous hyperalgesia: the effects of interleukin-10 and glial inhibitors. Mol. Pain.

[30] El Karim IA (2011). Human dental pulp fibroblasts express the “cold-sensing” transient receptor potential channels TRPA1 and TRPM8. J. Endod..

[31] Andrew D, Matthews B (2000). Displacement of the contents of dentinal tubules and sensory transduction in intradental nerves of the cat. J. Physiol..

[32] Charoenlarp P, Wanachantararak S, Vongsavan N, Matthews B (2007). Pain and the rate of dentinal fluid flow produced by hydrostatic pressure stimulation of exposed dentine in man. Arch. Oral Biol..

[33] Munns C (2007). Al Qatari, M. & Koltzenburg, M. Many cold sensitive peripheral neurons of the mouse do not express TRPM8 or TRPA1. Cell Calcium.

[34] Memon T, Chase K, Leavitt LS, Olivera BM, Teichert RW (2017). TRPA1 expression levels and excitability brake by KV channels influence cold sensitivity of TRPA1-expressing neurons. Neuroscience.

[35] Thut PD, Wrigley D, Gold MS (2003). Cold transduction in rat trigeminal ganglia neurons *in vitro*. Neuroscience.

[36] Noël J (2009). The mechano-activated K+ channels TRAAK and TREK-1 control both warm and cold perception. EMBO J..

[37] Yin K, Zimmermann K, Vetter I, Lewis RJ (2015). Therapeutic opportunities for targeting cold pain pathways. Biochem. Pharmacol..

[38] Dhaka A (2007). TRPM8 is required for cold sensation in mice. Neuron.

[39] Dong, H. W. The Allen Reference Atlas: A Digital Color Brain Atlas of the C57BL/6J Male Mouse. John Wiley & Sons Inc. (2008).

[40] Malin SA, Davis BM, Molliver DC (2007). Production of dissociated sensory neuron cultures and considerations for their use in studying neuronal function and plasticity. Nat. Protoc..

