# Supplementary material for: TRPM8 and TRPA1 do not contribute to dental pulp sensitivity to cold
Source: Sci Rep. 2018 Sep 4;8:13198. doi: 10.1038/s41598-018-31487-2 (PMC6123413; doi:10.1038/s41598-018-31487-2)
Supplement: Supplementary file 1 — Supplementary Figures [file 41598_2018_31487_MOESM1_ESM.pdf]

TRPM8 and TRPA1 do not contribute to dental pulp sensitivity to cold

Benoit Michot*, Caroline S. Lee, Jennifer L. Gibbs

Department of Endodontics, New York University College of Dentistry, New York, USA.

* Corresponding author:
Benoit Michot
New York University – College of Dentistry
345 E. 24th Street
New York, NY 10010
Email : bm119@nyu.edu; bemichot@yahoo.fr

Supplementary Figure S1

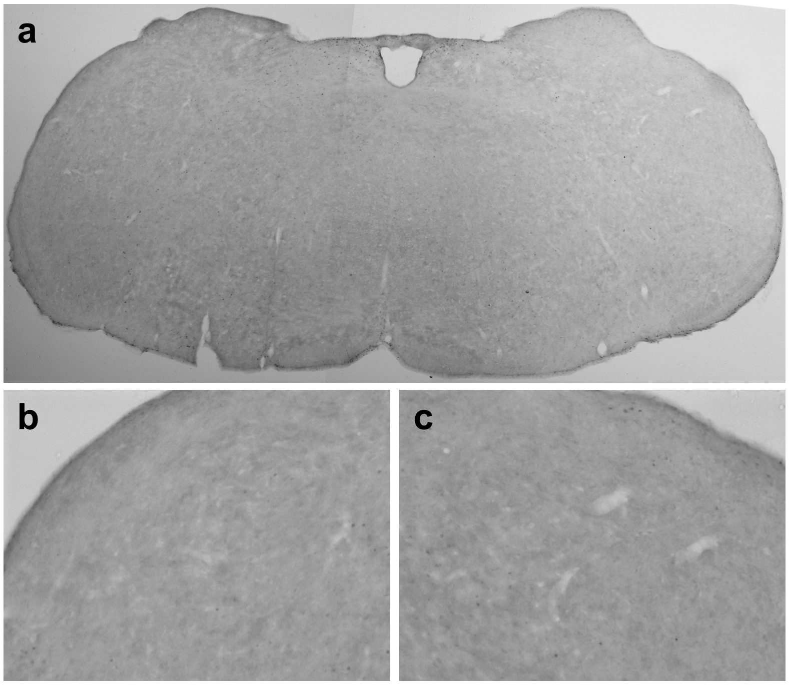

Figure S1: Representative photo-micrographs of cFos immunostaining in the Vi/Vc transition zone in mice that underwent a repeated unilateral light mechanical stimulation of the maxillary first molar with cotton (15 applications over a 30-min period). a) shows a low magnification image of the trigeminal nucleus and b) and c) show a high magnification images of the dorsolateral trigeminal nucleus contralateral and ipsilateral to the stimulation side respectively.
